# Serotonin and sleep-promoting neurons

**DOI:** 10.18632/oncotarget.13419

**Published:** 2016-11-16

**Authors:** Armelle Rancillac

**Affiliations:** Center for Interdisciplinary Research in Biology (CIRB), CNRS UMR 7241 / Inserm U1050, Collège de France, Paris, France

**Keywords:** VLPO, slow-wave sleep (SWS), non-rapid eye movement sleep (NREM), 5-HT2C receptor, 5-HT1A receptor

Serotonin (5-HT) is a monoamine neurotransmitter that plays major roles in several physiological functions including circadian rhythmicity, thermoregulation, emotion, cognition and nociception. The relationship between 5-HT and sleep was demonstrated by several experiments. In a particularly elegant one, cats were rendered insomniac following the injection of a 5-HT synthesis inhibitor. In this model, the preoptic area of the hypothalamus containing the ventrolateral preoptic area (VLPO) was the only brain region in which microinjections of the 5-HT precursor could restore long periods of sleep [1]. The VLPO being the main brain structure inducing slow-wave sleep (SWS), it was then hypothesized that 5-HT modulation of neuronal activity in this structure is essential for sleep regulation. However, the mechanisms involved in the physiological role of 5-HT within the VLPO remained largely unknown.

The VLPO is a small cluster of neurons composed of different neuronal populations. Five distinct neuronal classes were recently electrophysiologically defined [2]. These neuronal populations were morphologically characterized and were shown to differently respond to neurotransmitters such as noradrenaline (NA) and 5-HT. Indeed, sleep-promoting neurons within the VLPO are usually identified by their inhibitory response to NA, suggesting that they are maintained silent during waking. They are either inhibited (Type-1) or excited (Type-2) by 5-HT application [3].

In our paper, the mode of action and the effects of 5-HT were determined in Type-1 and Type-2 sleep-promoting neurons. In acute VLPO slices of mouse, spontaneous and miniatures excitatory and inhibitory postsynaptic currents were recorded in response to bath application of 5-HT. We found that 5-HT reduces frequencies of all these events to Type-1 neurons, whereas 5-HT selectively increases the frequencies of inhibitory events to Type-2 VLPO neurons [4].

Furthermore, our data shows that Type-1 and Type-2 sleep-promoting neurons present similar morphological somatic hallmarks as measured on infrared microphotography taken prior electrophysiological recordings. However, we established that the area of Type-1 neurons was significantly smaller compared to Type-2 neurons. Membrane properties of sleep-promoting neurons were also investigated eletrophysiologically. We found that the action potential threshold was significantly lower in Type-1 compared to Type-2 neurons. As Type- 2 neurons have been previously shown to selectively respond to an A_*2A*_ adenosine receptor agonist [3, 5], they could likely integrate the homeostatic drive associated with adenosine accumulation during wakefulness, and first respond to the serotonergic excitatory inputs. Then, the activation of Type-2 neurons would decrease the frequency of GABAergic inputs to Type-1 neurons and favor their activation. Type-1 VLPO neurons would then inhibit arousal systems and allow the maintenance of slow-wave sleep.

Finally, we investigated the molecular diversity of sleep-promoting neurons using the single-cell RT-PCR technique to simultaneously detect the expression of the 13 serotonergic receptors. We established that *5-HT_1_* receptors mRNA were only detected in Type-1 neurons, whereas mRNAs encoding *5-HT_2A-C_*, *5-HT_4_* and *5-HT_7_* were equally distributed between Type-1 and Type- 2 VLPO neurons. To confirm the putative expression of 5-HT_*1A*_ and 5-HT_*2C*_ receptors that were the most frequently amplified mRNA, we applied potent agonist of these receptors on sleep-promoting neurons. In loose-patch recordings, we established that the selective 5-HT_*1A*_ receptor agonist, the 8-OH-DPAT, decreases the firing frequency of Type-1 neurons, whereas a 5-HT2C receptor agonist, PF03246799, enhances the firing frequency of Type-2 neurons. Interestingly, we also observed that the decreased firing rate induced by 5-HT application in Type- 1 neurons was subsequently followed by a small increased of their firing rate and that PF03246799 application could also increase the firing rate in a subset of Type-1 neurons. Physiologically, we hypothesize that afferent serotonergic inputs to the VLPO could exert a complex and fine control of sleep-promoting neurons. Nevertheless, it appears from the literature that systemic injections of a 5-HT agonist could produce opposite effects on sleep amounts, depending on the concentration used and on the time of the sleep-wake cycle during which the treatment was administered [6, 7]. These studies suggest a circadian modulation of serotonergic receptor function.

**Figure 1:**
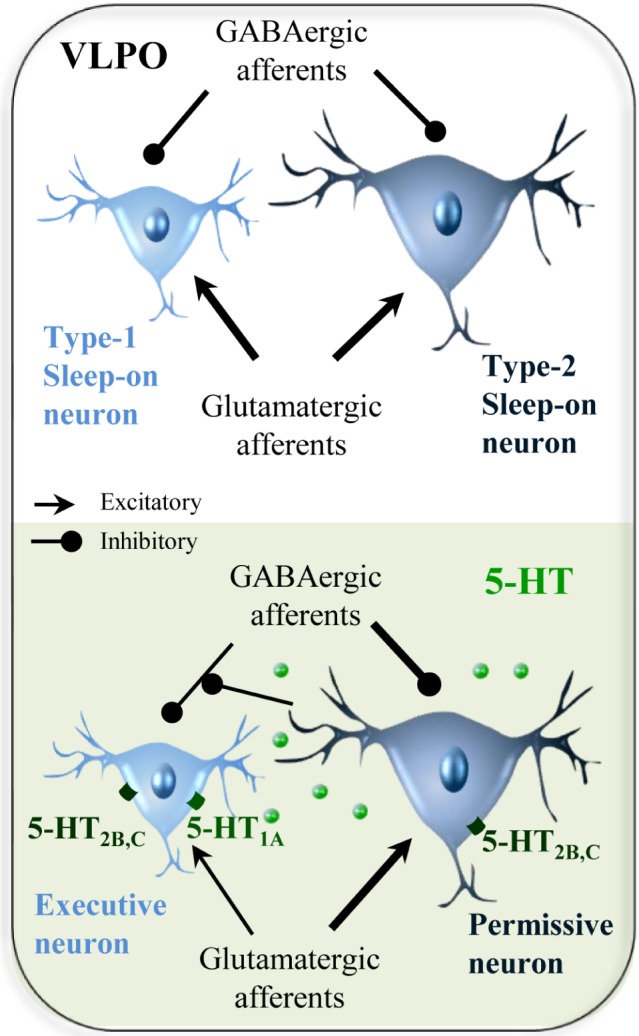
Schematic drawing depicting the potential mechanism of the regulation of afferent inputs to sleep-promoting neurons by 5-HT

Altogether, our results established electrophysiological, morphological and molecular differences between these two neuronal types of sleep-promoting neurons. Type-2 neurons being more excitable, they could likely integrate the homeostatic drive of sleep and be involved in the preparation and initiation of sleep (permissive neurons). Then, their activation could decrease the frequency of inhibitory inputs to Type-1 neurons to favor their activation. Type-1 VLPO neurons would then inhibit arousal systems and allow the maintenance of SWS (executive neurons, Figure 1).

Our work provides new insights regarding sleep regulation by 5-HT and propose distinct roles for Type- 1 and Type-2 neurons. Furthermore, we established that sleep-promoting neurons frequently express 5-HT_*2C*_ receptors that could represent a molecular target for the development of safer and more effective sleep-promoting medication to treat insomnia and to improve quality of life.
